# Effect of Body Mass Index on Outcomes of Percutaneous Nephrolithotomy: A Systematic Review and Meta-Analysis

**DOI:** 10.3389/fsurg.2022.922451

**Published:** 2022-06-14

**Authors:** Yan Xu, Xiaolin Huang

**Affiliations:** Outpatient Operating Room, Wenzhou Hospital of Integrated Traditional Chinese and Western Medicine, Wenzhou, China

**Keywords:** obesity, overweight, renal stones, surgery, complications, nephrolithiasis

## Abstract

**Objective:**

The current study aimed to assess the efficacy and safety of percutaneous nephrolithotomy (PCNL) in obese and overweight individuals based on body mass index (BMI).

**Methods:**

We electronically explored the databases of PubMed, CENTRAL, ScienceDirect, Embase, and Google Scholar databases for all types of comparative studies investigating the role of BMI on PCNL outcomes. Only studies defining obesity as >30 kg/m^2^ were included. Efficacy outcomes were stone-free rates and operating time while safety outcomes were complications and length of hospital stay (LOS).

**Results:**

Eighteen studies with 101,363 patients were included. We noted no difference in the stone-free rates after PCNL for morbid obese vs normal BMI patients (OR: 0.78 95% CI, 0.57, 1.08 I^2 ^= 7% *p* = 0.13), overweight vs normal (OR: 1.01 95% CI, 0.89, 1.15 I^2 ^= 1% *p* = 0.83) and obese vs normal patients (OR: 1.00 95% CI, 0.87, 1.16 I^2 ^= 0% *p* = 0.95). PCNL operative time was significantly increased in morbid obese (MD: 9.36 95% CI, 2.85, 15.88 I^2 ^= 76% *p* = 0.005) and obese patients as compared with normal patients (MD: 2.15 95% CI, 1.20, 3.10 I^2 ^= 0% *p* < 0.00001), but not for overweight patients. There was no difference in the odds of complications between morbid obese vs normal (OR: 1.26 95% CI, 0.93, 1.72 I^2 ^= 0% *p* = 0.13), overweight vs normal (OR: 1.11 95% CI, 0.96, 1.28 I^2 ^= 0% *p* = 0.15), and obese vs normal patients (OR: 1.07 95% CI, 0.91, 1.27 I^2 ^= 0% *p* = 0.40). LOS was significantly reduced in obese patients (MD: −0.12 95% CI, −0.20, −0.04 I^2 ^= 0% *p* = 0.004) as compared to normal patients, but not for morbid obese or overweight patients.

**Conclusion:**

PCNL has similar efficacy and safety in morbidly obese, obese, and overweight patients as compared to normal BMI patients with no difference in the stone-free and complication rates. Evidence suggests that operating time is increased in morbidly obese and obese patients and the latter may have shorter LOS.

**Systematic Review Registration:** https://www.crd.york.ac.uk/prospero/, identifier: CRD42022313599.

## Introduction

Renal stones are a very common urological disease that has a significant impact on the health and quality of life of affected patients (1). The prevalence of the disease varies from 0.1 to 14.8% amongst Western populations to as high as 10.6% in the Asian population (1, 2). Indeed, renal stones can lead to significant patient morbidity causing symptoms like abdominal pain, infections, hydronephrosis, and decreased renal function (1). It is well known that colicky pain due to renal stones is one of the most common presenting symptoms in the emergency department (3). Management protocols for renal stones include observation and pharmacotherapy or invasive procedures like shockwave lithotripsy, percutaneous nephrolithotomy (PCNL), ureterorenoscopy, and even laparoscopic/open renal surgeries for severe cases (4).

As with the high prevalence of renal stones, the world is also witnessing an obesity epidemic. The worldwide prevalence of obesity has exponentially increased from <1% in 1975 to 6%–8% in 2016 (5). The prevalence, however, varies significantly across countries ranging from 0.2% in Vietnamese women to as high as 65.3% in American Samoan women (6). With such growing incidence, a large number of obese patients are being seen by a urologist for managing renal stone disease. Management of such patients is challenging not only due to the change in body habitus but also due to the presence of other comorbidities like diabetes, hypertension, coronary heart disease, atrial fibrillation, and heart failure (7). Obesity restricts the application of shockwave lithotripsy due to the increased patient weight, problems with stone localization, and increased skin to stone distance (8). Delekas et al. (9) in a cohort study of 502 patients have shown that obesity is an independent predictor of failure after shockwave lithotripsy. Ureterorenoscopy is considered to be a safe and efficacious procedure for obese patients but has disadvantages of increased operating time based on the size, location, and the number of calculi (10). Its success rate also depends on the experience of the operating surgeon (11). On the other hand, PCNL is highly efficacious in treating patients with stones of >2cms in size, multiple calculi, and staghorn calculi (12). PCNL can achieve high stone-free rates as compared to shockwave lithotripsy and has significantly lower morbidity as opposed to open surgery (13). However, it is unclear if PCNL has similar efficacy and safety in obese patients as compared to normal body mass index (BMI) patients. In the past, Zhou et al. (14) in their systematic review have attempted to compare outcomes of obese and normal BMI patients undergoing PCNL. Using the WHO based definitions of normal (<25 kg/m^2^), overweight (25–29.9 kg/m^2^), obese (30–39.9 kg/m^2^), and morbid obese (≥40 kg/m^2^), the authors concluded that obesity had no impact on the outcomes of PCNL except for longer operating times in morbid obese and shorter hospital stay in obese patients. However, their review could include just seven studies. With the publication of several new studies in the recent past, there is a need for high-quality updated evidence to guide urologists on the clinical efficacy of PCNL for obese patients. We therefore conducted the current study to investigate the role of BMI on the efficacy and safety of PCNL by means of a systematic review and meta-analysis.

## Material and Methods

### Search Strategy

The online systematic review database of PROSPERO was used to register our meta-analysis (CRD42022313599). We carried out a systematic and comprehensive electronic database search of the PubMed, CENTRAL, ScienceDirect, Embase, and Google Scholar databases. Since Google Scholar has a large number of search records, we restricted the search to only the initial 100 results of each query. The last day of the search was 1^st^ March 2022. Results were limited to English-language studies. The search terms used were: “percutaneous nephrolithotomy”, “nephrolithiasis”, “urolithiasis”, “renal stones”, “body mass index”, “BMI”, “obese”, “obesity”, “overweight”, and “treatment” (Supplementary Table S1). The search results were imported in a reference manager and deduplicated. The reviewers carried out an initial screening using the title and abstracts of the search results. Only relevant publications were downloaded. The two reviewers then independently cross-checked each study against the eligibility criteria for final inclusion in our review. Any disagreements were cleared by consensus. The bibliography of studies included were then hand-searched to find any other possible inclusions. We also adhered to the PRISMA statement (Preferred Reporting Items for Systematic Reviews and Meta-analyses) during the reporting of the review (15).

### Inclusion/Exclusion

The following studies were included (Table 1): (1) All types of comparative studies conducted on adult patients undergoing PCNL for renal stones with no restriction on study design. (2) Studies comparing data of patients based on BMI. (3) Studies were to define obese patients as BMI of >30 kg/m^2^. (4) Studies were to report any of the following outcomes: stone-free rates, operation time, complications, and/or length of hospital stay (LOS).

**Table 1:** PICOS inclusion criteria of the review.

Domain	Review criteria
Population	Adult patients undergoing PCNL for renal stones
Intervention	Obesity (defined as ≥30 kg/m^2^)
Comparison	Non-obese (defined as <30 kg/m^2^) or Normal BMI (defined as <25 kg/m^2^)
Outcomes	Stone-free rates, operation time, complications, and/or length of hospital stay
Study types	All comparative study designs

BMI, body mass index; PCNL, percutaneous nephrolithotomy.

Exclusion criteria were: (1) Studies reporting the combined effect of PCNL with any other surgical treatment modality (2) Not defining obese patients as BMI of ≥30 kg/m^2^ (3) Studies reporting duplicate data from the same database. If there was a partial overlap of data from two studies, the article with a greater sample size was to be included.

### Data Management and Risk of Bias

Data extraction was carried out by two reviewers independent of each other using a standardized data collection form. Data extracted included author details, publication year, study type, study location, the database used, study groups, BMI definition, sample size, demographic details, stone size, the definition of stone-free status, patient position, sheath and nephroscope used, type of lithotripsy, diagnostic modality for stone-free status, follow-up period and outcomes. We did not define stone-free rates a priori and accepted all definitions used by the included studies. The outcomes of interest were: stone-free rates, operating times, complications, and LOS.

Two reviewers examined the quality of studies using the Newcastle-Ottawa scale (NOS) (16). Any disagreements between the reviewers were cleared by consensus. The NOS tool has the following domains: selection of study population, comparability, and outcomes. A maximum of four, two, and three points are allocated to each domain in this respective order. The highest score for a study can be nine.

### Statistical Analysis

Dichotomous data were combined using odds ratios (OR) and continuous data were combined using mean difference (MD) with 95% confidence intervals (CI) in a random-effects model. We performed separate comparisons for morbid obese (BMI ≥40 kg/m^2^) vs normal (BMI <25 kg/m^2^), obese (BMI ≥30 kg/m^2^) vs normal, overweight (BMI 25–29.9 kg/m^2^) vs normal, and obese vs non-obese patients (BMI ≥30 kg/m^2^ vs <30 kg/m^2^).

Inter-study heterogeneity was examined by the I^2^ statistic. I^2^ values were classified as 25–50% = low, 50–75% = medium, and >75% = substantial heterogeneity. *p* values of <0.05 were considered statistically significant. We used “Review Manager” (RevMan, version 5.3; Nordic Cochrane Centre [Cochrane Collaboration], Copenhagen, Denmark; 2014) for the meta-analysis. Publication bias was examined by means of funnel plots. A leave-one-out sensitivity analysis was done by excluding one study at a time and assessing the results again. However, this was done only for analyses including more than three studies.

## Results

### Search Results

**Figure 1:**
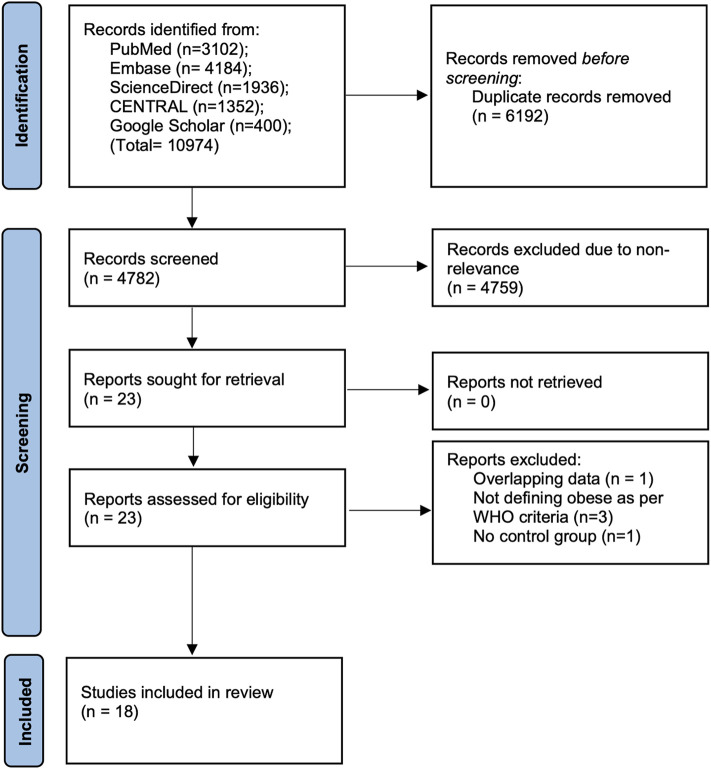
PRISMA flow-chart of the study.

We identified a total of 4,782 unique articles (Figure 1). On initial titles and abstract screening, 4,759 articles were excluded due to non-relevance. The authors selected 23 articles for full-text analysis. Of these, 5 were excluded due to various reasons. A total of 18 studies were then included in the current review (17–34).

All studies were prospective or retrospective observational studies published between 2004 to 2020 (Table 2). Four studies compared data between obese and non-obese patients only. The remaining studies divided the cohort into multiple groups of morbidly obese, obese, overweight, and normal. The study of Shohab et al. (20) defined obesity based on WHO classification but used the cut-off of 24 kg/m^2^ to separate overweight and normal BMI patients. Since this was a minor change, the study was included in the meta-analysis. A few studies (24, 29, 32) also deviated from the WHO definition of morbidly obese and defined it as ≥35 kg/m^2^. However, since the definition of obese and overweight was based on WHO criteria these studies were included in the analysis but not in the subgroup analysis of morbid obese vs normal. The included studies examined a data of 101,363 patients. The NOS score ranged from 6 to 9. PCNL was performed in the prone position in most of the studies (Table 3). Only two studies reported the use of supine position. The majority of the studies did not define the cut-off for stone-free status. The definition was provided by just six studies that used <4 mm, ≤4 mm, or ≤3 mm as the cut-off for stone-free status.

**Table 2:** Details of included studies.

Study	Location	Database	Groups	BMI definition (kg/m^2^)	Sample size	Mean age (Years)	Male gender (%)	Mean stone size (mm)	Follow-up	NOS score
Ferreira 2020 (26)	Brazil	Hospital Brigadeiro	Obese	≥30	94	48.5 ± 10.5	32.7	26.1 ± 9.1	Immediate	7
			Non-Obese	<30	307	48.1 ± 13.4	49.6	28.1 ± 10.2		
Desoky 2020 (25)	Egypt	Zagazig University	Morbid obese	≥40	116	46.1 ± 12.9	47.4	38 ± 9.3	1 month	7
			Obese	30–39.9	116	46.2 ± 12.7	55.2	38.8 ± 10.1		
			Overweight	25–29.9	116	46 ± 12.9	61.2	39.1 ± 10.1		
			Normal	18.5–24.9	116	45.9 ± 13.1	59.5	39.2 ± 10.1		
Jin 2019 (24)	China	Sheng Jing Hospital	Morbid obese	≥35	21	52.7 ± 10.2	42.9	NR	2 days	7
			Obese	30–34.9	75	47.2 ± 7.9	50.7			
			Overweight	25–29.9	166	51 ± 9.8	53.6			
			Normal	<25	97	48.7 ± 8.7	58.8			
Isoglu 2017 (23)	Turkey	Tepecik Education and Research Hospital	Obese	≥30	282	48.9 ± NR	53.2	NR	1 month	7
			Non-obese	<30	676	47.9 ± NR	62.4			
Usawachintachit 2016 (22)	USA & Thailand	University of California & King Chulalongkorn Memorial Hospital	Obese	≥30	35	37.9 ± 9	40	34.6 ± 15.9	1 day	7
			Overweight	25–29.9	19	26.7 ± 1.5	52.6	23.3 ± 12.1		
			Normal	<25	39	21.4 ± 2.5	35.9	36.9 ± 29.1		
Trudeau 2016 (21)	USA	Nationwide Inpatient Sample	Obese	≥30	9300	51.9 ± 12.6	36.7	NR	NR	6
			Non-obese	<30	81229	52.8 ± 15.5	49.6			
Akbulut 2016 (19)	Turkey	Haseki Training and Research Hospital	Obese	≥30	49	51.7 ± 12.5	44.9	26.2 ± 8.6	1 month	7
			Non-obese	<30	133	41.8 ± 12.6	31.6	23.9 ± 9		
Shohab 2015 (20)	Pakistan	Shifa International Hospitals	Obese	>30	26	43.6 ± 1.3	NR	26.8 ± 7.4	NR	7
			Overweight	24.1–30	56	47.1 ± 1.3		28 ± 8.4		
			Normal	≤24	47	43.3 ± 1.7		25.5 ± 8.9		
Simsek 2014 (34)	Turkey	Haseki Training and Research Hospital	Morbid obese	≥40	36	50.2 ± 11.1	55.6	NR	Immediate	7
			Obese	30–39.9	334	49.5 ± 12.8	64.9			
			Overweight	25–29.9	883	46.4 ± 12.9	57.8			
			Normal	<25	849	38.2 ± 14.8	57.7			
Ortiz 2014 (33)	Spain	Hospital Universitario de Bellvitge	Morbid obese	≥40	10	58.4 ± 11.2	30	NR	NR	7
			Obese	30–39.9	75	54.7 ± 12.1	53			
			Overweight	25–29.9	93	56.2 ± 13.3	61			
			Normal	<25	77	51.9 ± 15.8	53			
Kuntz 2013 (32)	USA	Duke University Medical Center	Morbid obese	≥35	72	NR	43	NR	3 months	9
			Obese	30–34.9	67		52			
			Overweight	25–29.9	74		50			
			Normal	<25	55		40			
Alyami 2013 (31)	Canada	Dalhousie University	Morbid obese	≥40	10	53 ± 4.1	50	24 ± 3.9	NR	7
			Obese	30–39.9	41	60 ± 2.2	36.6	22 ± 1.6		
			Overweight	25–29.9	24	60 ± 2.8	41.7	23 ± 2		
			Normal	<25	39	55 ± 2.6	58.9	23 ± 1.4		
Fuller 2012 (30)	Multi-national	CROES database	Morbid obese	≥40	97	55.3 ± 12.1	33	NR	NR	6
			Obese	30–39.9	650	55.5 ± 12.9	51.5			
			Overweight	25–29.9	1568	52.2 ± 13.3	61.9			
			Normal	<25	1394	46.1 ± 14.9	54.2			
Tomaszewski 2010 (29)	USA	University of Pittsburgh School of Medicine	Morbid obese	≥35	38	NR	NR	39 ± 20	NR	7
			Obese	30–34.9	43			37 ± 20		
			Overweight	25–29.9	45			31 ± 12		
			Normal	<25	61			36 ± 19		
Bagrodia 2008 (28)	USA	University of Texas Southwestern Medical Center	Morbid obese	≥40	29	45 ± NR	NR	23 ± NR	Immediate	7
			Obese	30–39.9	51	53 ± NR		18 ± NR		
			Overweight	25–29.9	44	54 ± NR		16 ± NR		
			Normal	<25	26	58 ± NR		17 ± NR		
Sergeyev 2007 (27)	USA	Albert Einstein College of Medicine	Obese	≥30	37	52.5 ± 12.4	NR	NR	2 days	8
			Overweight	25–29.9	33	52.8 ± 13				
			Normal	<25	15	57.9 ± 18.8				
El-Assmy 2007 (18)	Egypt	Mansoura University	Morbid obese	≥40	92	46.5 ± 10	46.7	25 ± 8	3 months	7
			Obese	30–39.9	468	46.9 ± 10.5	64.5	24 ± 8		
			Overweight	25–29.9	325	47 ± 10.9	67.7	25 ± 7		
			Normal	<25	270	46.5 ± 10.9	65.2	25 ± 7		
Koo 2004 (17)	UK	Norfolk and Norwich University Hospital	Morbid obese	≥40	12	51 ± 14	50	NR	4–8 weeks	7
			Obese	30–39.9	67	56 ± 14	82.1			
			Overweight	25–29.9	79	56 ± 15	68.4			
			Normal	<25	65	50 ± 18	53.8			

*BMI, Body mass index; CROES, Clinical Research Office of the Endourological Society; NR, not reported.*

**Table 3:** Procedural details from the included studies.

Study	Position	Sheath	Nephroscope used	Lithotripsy method	Cut-off for stone-free status (mm)	Follow-up imaging
Ferreira 2020 (26)	Supine	30 Fr Alken	NR	Ultrasonic	≤4	CT
Desoky 2020 (25)	Supine	NR	NR	NR	<4	CT
Jin 2019 (24)	Prone	24 Fr Alken	20.8 Fr	Ultrasonic and pneumatic	<4	CT
Isoglu 2017 (23)	Prone	28–30 Fr Amplatz	24 Fr	Ultrasonic	≤4	CT
Usawachintachit 2016 (22)	Prone	NR	NR	NR	NR	X-ray KUB, USG and fluoroscopic imaging
Trudeau 2016 (21)	NR	NR	NR	NR	NR	NR
Akbulut 2016 (19)	Prone	18 Fr Amplatz	12 or 17 Fr	Ultrasonic or pneumatic or laser	<4	X-ray KUB, USG or CT
Shohab 2015 (20)	Prone	30 Fr Amplatz	26 Fr	Pneumatic	NR	X-ray KUB, USG or CT
Simsek 2014 (34)	Prone	NR	26 Fr	Ultrasonic	NR	X-ray KUB
Ortiz 2014 (33)	Prone	NR	NR	NR	NR	NR
Kuntz 2013 (32)	NR	NR	NR	NR	NR	CT or IVU
Alyami 2013 (31)	Prone	30 Fr Amplatz	NR	Ultrasonic or pneumatic	NR	NR
Fuller 2012 (30)	Supine	NR	NR	Ultrasonic or pneumatic or laser	NR	NR
Tomaszewski 2010 (29)	Prone	NR	NR	Ultrasonic or pneumatic or laser	0	X-ray KUB, USG, CT
Bagrodia 2008 (28)	NR	NR	NR	NR	NR	CT
Sergeyev 2007 (27)	Prone	30 Fr Amplatz	NR	Ultrasonic	≤3	X-ray KUB or CT
El-Assmy 2007 (18)	Mostly prone (93.3%)	30 Fr Amplatz	NR	Ultrasonic or pneumatic	NR	X-ray KUB, USG, CT
Koo 2004 (17)	Prone	30 Fr Amplatz	NR	Pneumatic	NR	X-ray KUB

*CT, Computed tomography; Fr, French; IVU; Intravenous urography; KUB, Kidney urinary bladder; NR, not reported.*

### Stone-free Rates

**Figure 2:**
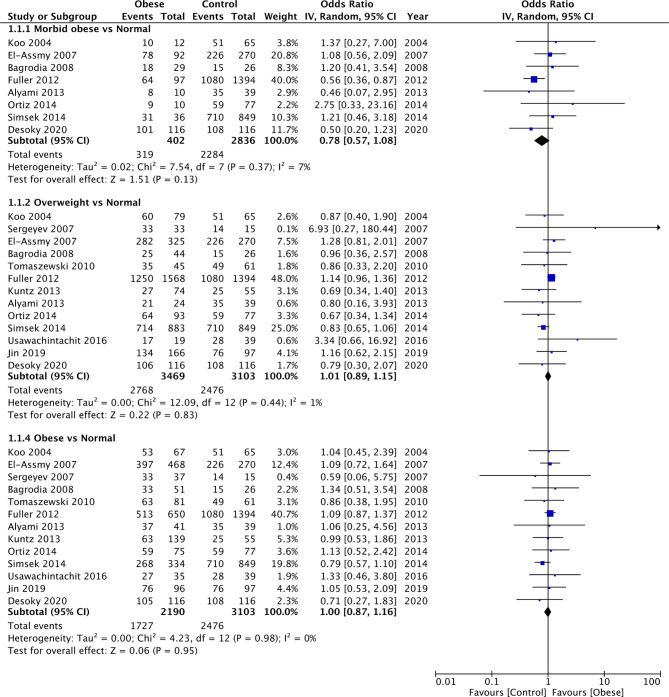
Meta-analysis of stone-free rates between morbid obese, overweight and obese vs normal BMI patients.

**Figure 3:**
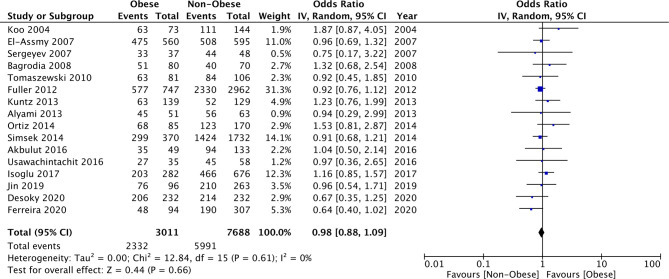
Meta-analysis of stone-free rates between obese vs non-obese patients.

Comparing data of morbid obese vs normal BMI patients, our meta-analysis failed to demonstrate any statistically significant difference in stone-free rates between the two groups (OR: 0.78 95% CI, 0.57, 1.08 I^2 ^= 7% *p* = 0.13) (Figure 2). Similarly, we also noted no difference in the stone-free rates after PCNL for overweight vs normal (OR: 1.01 95% CI, 0.89, 1.15 I^2 ^= 1% *p* = 0.83) and obese vs normal patients (OR: 1.00 95% CI, 0.87, 1.16 I^2 ^= 0% *p* = 0.95) as well (Figure 2). On comparison of data of obese vs non-obese patients with the cut-off of 30 kg/m^2^, we noted no difference in the stone-free rates after PCNL (OR: 0.98 95% CI, 0.88, 1.09 I^2 ^= 0% *p* = 0.66) (Figure 3). There was no evidence of publication bias (Supplementary Figure S1).

The results of all comparisons were stable on sensitivity analysis with no change in the significance, except for the comparison of morbid obese vs normal. On the exclusion of the study of El-Assmy et al. (18), the results indicated lower stone-free rates in morbid obese vs normal BMI patients (OR: 0.70 95% CI, 0.50, 0.99 I^2 ^= 2% *p* = 0.04).

### Operation Time

**Figure 4:**
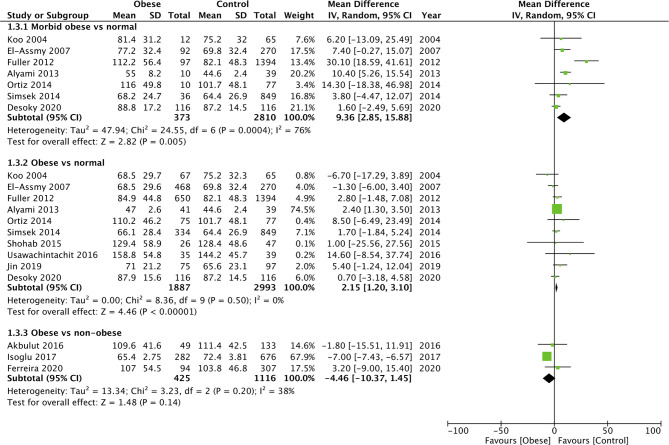
Meta-analysis of operation time between morbid obese vs normal, Obese vs normal, and obese vs non-obese patients.

**Figure 5:**
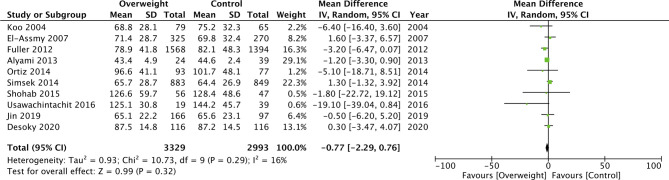
Meta-analysis of operation time between overweight vs normal patients.

Our meta-analysis demonstrated that PCNL operative time was significantly increased in morbidly obese patients as compared to normal patients (MD: 9.36 95% CI, 2.85, 15.88 I^2 ^= 76% *p* = 0.005) (Figure 4). A similar difference was noted when obese patients were compared with normal patients (MD: 2.15 95% CI, 1.20, 3.10 I^2 ^= 0% *p* < 0.00001) (Figure 4). However, on a pooled analysis of three studies, comparing data of obese vs non-obese patients, we noted no difference in PCNL operating time between the two groups (MD: −4.46 95% CI, −10.37, 1.45 I^2 ^= 38% *p* = 0.14) (Figure 4). We also noted no statistically significant difference in the operating time between overweight vs normal patients (MD: −0.77 95% CI, −2.29, 0.76 I^2 ^= 16% *p* = 0.32) (Figure 5).

For the comparison of obese vs normal patients, the results turned non-significant on the exclusion of the study of Alyami et al. (31) (MD: 1.42 95% CI, −0.45, 3.29 I^2 ^= 0% *p* = 0.14). However, the results of the analysis of morbid obese vs normal patients and overweight vs normal patients were stable on sensitivity analysis.

### Complications

**Figure 6:**
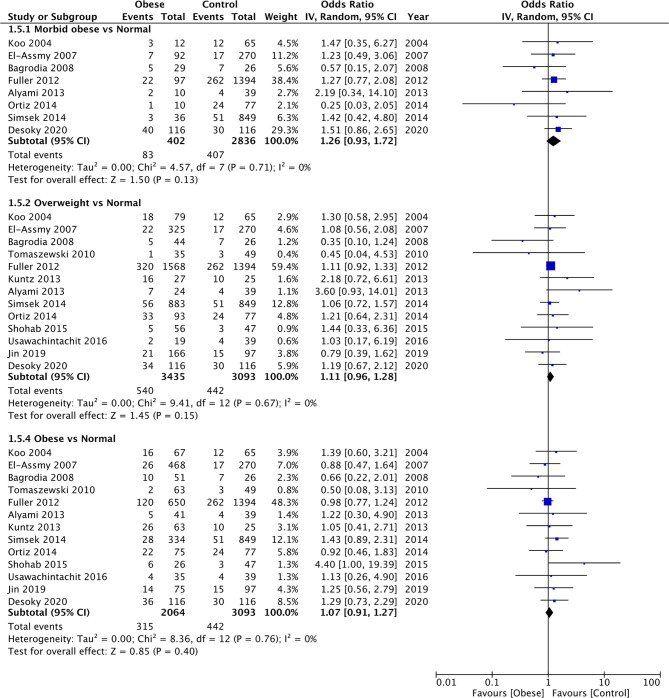
Meta-analysis of complication rates between morbid obese, overweight and obese vs normal BMI patients.

**Figure 7:**
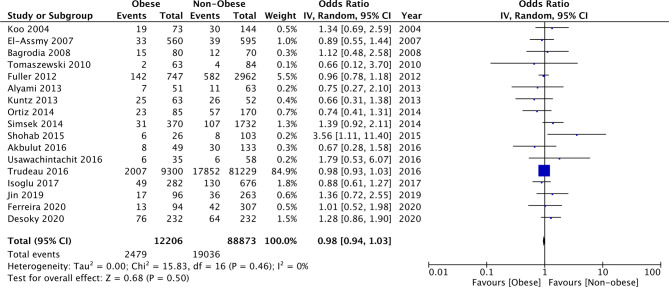
Meta-analysis of complication rates between obese vs non-obese patients.

Since several different complications were described in the studies, separate analyses for the same could not be conducted. Our meta-analysis indicated no difference in the odds of complications between morbid obese vs normal (OR: 1.26 95% CI, 0.93, 1.72 I^2 ^= 0% *p* = 0.13), overweight vs normal (OR: 1.11 95% CI, 0.96, 1.28 I^2 ^= 0% *p* = 0.15), and obese vs normal patients (OR: 1.07 95% CI, 0.91, 1.27 I^2 ^= 0% *p* = 0.40) (Figure 6). Similarly, on comparing data of obese vs non-obese patients, we noted no difference in complication rates between the two groups (OR: 0.98 95% CI, 0.94, 1.03 I^2 ^= 0% *p* = 0.50) (Figure 7). We noted no evidence of publication bias on the funnel plot (Supplementary Figure S2). There was no change in the significance of the results on sensitivity analyses.

### LOS

**Figure 8:**
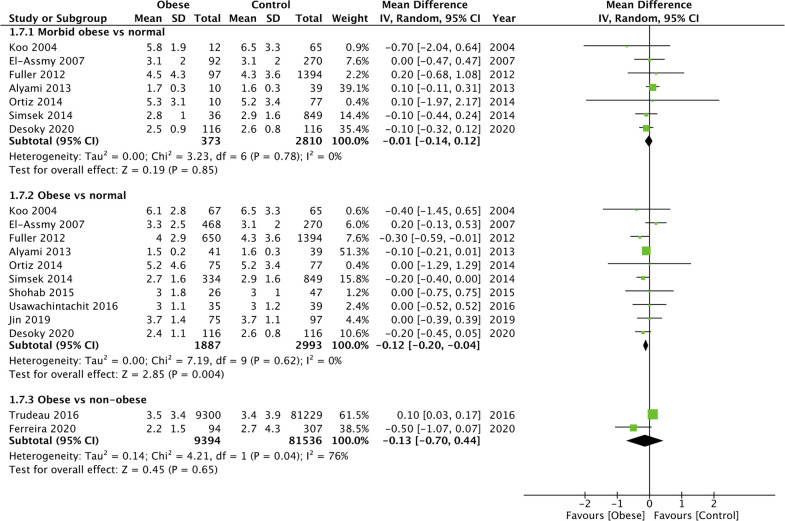
Meta-analysis of LOS between morbid obese vs normal, Obese vs normal and obese vs non-obese patients.

**Figure 9:**
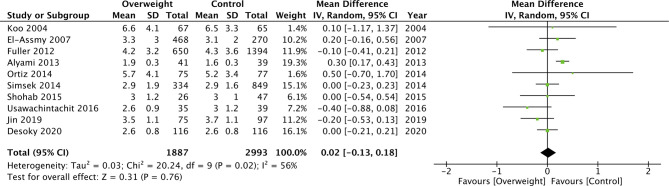
Meta-analysis of LOS between overweight vs normal patients.

On pooled analysis, we noted no statistically significant difference in the LOS between morbidly obese and normal BML patients undergoing PCNL (MD: −0.01 95% CI, −0.14, 0.12 I^2 ^= 0% *p* = 0.85) (Figure 8). However, the meta-analysis revealed that obese patients had significantly shorter LOS as compared to normal patients (MD: −0.12 95% CI, −0.20, −0.04 I^2 ^= 0% *p* = 0.004) (Figure 8). Combining data from two studies comparing obese vs non-obese patients, we noted no difference in the LOS between the two groups (MD: −0.13 95% CI, −0.70, −0.44 I^2 ^= 76% *p* = 0.65) (Figure 8). Similarly, we noted no difference in the LOS when analyzing data of overweight vs normal BMI patients (MD: 0.02 95% CI, −0.13, 0.18 I^2 ^= 56% *p* = 0.76) (Figure 9). All results were found to be stable on sensitivity analysis.

## Discussion

Overweight, obese, and morbidly obese patients represent a unique cohort of individuals who are difficult to manage by both physicians and surgeons (35). Indeed, these patients have several medical comorbidities which include cardiovascular, metabolic, and respiratory illnesses which can complicate the management of any other disease (36). Surgical procedures in these patients are frequently complicated and associated with an increased risk of complications with less than satisfactory outcomes (37, 38). Furthermore, research indicates that morbid obesity is an independent risk factor for mortality after surgery (36). Since obesity has become a major global healthcare problem, it is necessary to assess the impact of this comorbidity on every surgical procedure to enable risk stratification and informed clinical decisions. In this context, the current study comprehensively assessed the impact of morbid obesity, obesity, and overweight on outcomes of PCNL.

The success of any procedure performed for renal stones is measured by the stone-free status. Amongst the included studies, there was a wide variation in the stone-free rates after PCNL amongst obese and morbidly obese patients ranging from 62% to 90%. This is partly because of the non-standard definitions used by the included studies in assessing stone-free rates. Also, the majority of the studies did not report the cut-off used to assess residual fragments. Another difference was the imaging modality used by the studies to assess residual stones. While the recent studies used computed tomography (CT), older studies used either plain radiographs, ultrasonography, CT, or intravenous urography. It is well-known that different imaging modalities have different sensitivities and specificities to detect residual stones, with CT being the most efficient tool (39). However, since both obese and non-obese patients were assessed by the same criteria and imaging modality in each study, such differences may not have affected the overall outcomes of our review. Our meta-analysis demonstrated that morbidly obese, obese, and overweight individuals do not have worse stone-free rates as compared to normal BMI patients. Additionally, using the WHO-defined cut-off of 30 kg/m^2^, we also compared all obese patients (including morbid obese) vs non-obese patients (overweight and normal BMI) only to find no difference in the stone-free rates between the two groups. Despite the data being derived from observational studies, the lack of heterogeneity in the meta-analysis and lack of publication bias provides credibility to the results. Furthermore, most of the outcomes were stable on sensitivity analysis, and no study had a disproportionate effect on the results.

In the analysis of stone-free rates between morbid obese vs normal BMI patients, we noted that on the exclusion of one study, the results indicated inferior outcomes in morbidly obese patients. Importantly, this outcome was largely influenced by the results of Fuller et al. (30) which carried the maximum weight in the analysis (40%). Except for this study, all others reported no difference in stone-free rates between morbidly obese and normal BMI patients. In the study of Fuller et al. (30), the authors noted a significantly high rate of staghorn stones in morbid obese vs normal patients (40.2% vs 26%) which could have led to inferior outcomes in the obese cohort. Such disparity also points out another important factor in the interpretation of our results, which is, baseline differences between obese and normal BMI patients. It has been noted that diabetes, metabolic syndrome, and hypertension which are commonly seen with obesity are predisposing factors for renal stones (40). Obesity also leads to increased excretion of lithogenic substances, including calcium, oxalate, sodium, and uric acid thereby affecting urinary composition (41). Indeed, obese patients also have a higher incidence of uric acid stones (42). Insulin resistance associated with obesity may interfere with renal ammonium production and decrease urine pH (43). Thus, several factors can predispose an obese patient to renal stones and there could have been baseline differences between obese and normal BMI patients. In this context, the lack of propensity score matching for baseline variables is an important limitation of the included studies. However, even if it is assumed that obese patients had worse stone characteristics, the lack of difference in stone-free rates is indeed encouraging and PCNL should be routinely offered to such patients.

One of the difficulties in performing PNCL in an obese patient includes difficulty in the visualization of the stone under fluoroscopic or ultrasonographic guidance. The excess abdominal fat can hinder the identification of anatomical landmarks and stone positions making the procedure difficult. Standard instruments may also not be feasible for use in obese patients. Morbid obese patients require longer sheaths which can be difficult to handle and manipulate during the procedure. Indeed, we noted longer significantly longer operating times in morbidly obese and obese patients as compared to normal BMI patients. Our analysis demonstrated that operating time was increased by around 9 min for morbidly obese patients and 2 min for obese patients. The clinical significance of such a minor difference in operating times is indeed debatable.

Patient position is another important factor of consideration for obese patients. The majority of the included studies used the standard prone position for PCNL even for obese patients. Some studies have reported that obese patients in the prone position are vulnerable to abdominal compression due to intra-operative muscle relaxation which decreases total lung capacity and functional residual capacity. Furthermore, inferior vena cava compression can reduce preload and impair oxygenation leading to cardiopulmonary complications (30, 44). However, effect of patient position on cardiopulmonary complications during PCNL is conflicting and these is no clear evidence on what constitutes the best clinical practice. Nevertheless, to overcome such problems, researchers have advocated supine or lateral decubitus positioning, use of conscious sedation, and awake endotracheal intubation with prone patient self-positioning (45). Mazzucchi et al. (46) have compared outcomes of supine and prone PCNL in obese patients only to find no difference in clinical outcomes. Abouelleil et al. (47) have noted that the distance between skin to renal collecting system is significantly increased with supine as compared to the prone position and this difference increases with the patient’s BMI. The increased distance corresponds to increased nephrostomy tract length which reduces maneuverability and increases the difficulty of the procedure. There is also a possibility of higher surgical complications like bowel injury or bleeding from trauma to the kidney because of torquing of the renal parenchyma due to the larger distance (47). Further comparative studies are needed to provide clarity on what is the best position for obese patients undergoing PCNL.

Despite the majority of studies using the prone position, our meta-analysis failed to demonstrate any increased risk of complications in the overweight, obese, or morbid obese group. Even comparing obese vs non-obese patients, there was no difference in the risk of complications. It must be noted that all different complications reported by the studies were pooled in the meta-analysis. Only a limited number of studies classified the complication rates based on the Calvien-Dindo classification system, precluding a thorough analysis. We also noted no difference in the LOS in morbidly obese and overweight patients as compared to normal BMI patients. A small reduction in the LOS was noted in obese patients as compared to normal patients but with a MD of just 0.1 days. A difference of a few hours of hospital stay may not be clinically relevant.

There are certain limitations to our review. Firstly, all data was observational and therefore is prone to bias. Secondly, we were unable to assess the impact of important variables like patient positioning, stone size, stone composition, and stone location on the outcomes due to a lack of reporting of data by the included studies. Thirdly, we could not assess the risk of specific complications in the two groups. Incidence of specific complications would have produced better evidence. Fourthly, while all studies used the WHO criteria for defining obesity, the study of Shohab et al. (20) did not define overweight as per the WHO criteria. The small variation in definition could have resulted in bias in the analysis. However, we did not find any difference in outcomes on the exclusion of this study during sensitivity analysis. Lastly, the WHO cut-off for obesity is different for Asian and Non-Asian subjects. For Asian subjects, the WHO cut-off for obesity is 27 kg/m^2^. Therefore, our results may not be generalized for these patients till future studies report specific data on Asian population.

Nevertheless, our review has certain strengths. As compared to the previous review (14), we were able to add 11 more studies thereby significantly increasing the statistical power of the analyses. Unlike the previous review, we also compared outcomes of overweight and normal patients and obese vs non-obese patients.

## Conclusions

Our updated systematic review and meta-analysis of 18 observational studies including 101,363 participants have demonstrated that PCNL has similar efficacy and safety in morbidly obese, obese, and overweight patients as compared to normal BMI patients with no difference in the stone-free and complication rates. Evidence suggests that operating time is increased in morbidly obese and obese patients and the latter may have shorter LOS. Future studies should use propensity-score matching for baseline characteristics to increase the quality of evidence.

## Data Availability

Publicly available datasets were analyzed in this study. This data can be found here: The original contributions presented in the study are included in the article/Supplementary Material, further inquiries can be directed to the corresponding author/s.
