# Supplementary material for: Effect of Body Mass Index on Outcomes of Percutaneous Nephrolithotomy: A Systematic Review and Meta-Analysis
Source: Front Surg. 2022 Jun 14;9:922451. doi: 10.3389/fsurg.2022.922451 (PMC9237527; doi:10.3389/fsurg.2022.922451)
Supplement: Supplementary file 1 [file Table_1_v1.docx]

Supplementary Table 1: Search strategy

| **Query** | **Search Details** |
| --- | --- |
| (((((body mass index) OR (BMI)) OR (obese)) OR (obesity)) OR (overweight)) AND (percutaneous nephrolithotomy) | ("body mass index"[MeSH Terms] OR ("body"[All Fields] AND "mass"[All Fields] AND "index"[All Fields]) OR "body mass index"[All Fields] OR "BMI"[All Fields] OR ("obeses"[All Fields] OR "obesity"[MeSH Terms] OR "obesity"[All Fields] OR "obese"[All Fields] OR "obesities"[All Fields] OR "obesity s"[All Fields]) OR ("obeses"[All Fields] OR "obesity"[MeSH Terms] OR "obesity"[All Fields] OR "obese"[All Fields] OR "obesities"[All Fields] OR "obesity s"[All Fields]) OR ("overweight"[MeSH Terms] OR "overweight"[All Fields] OR "overweighted"[All Fields] OR "overweightness"[All Fields] OR "overweights"[All Fields])) AND ("nephrolithotomy, percutaneous"[MeSH Terms] OR ("nephrolithotomy"[All Fields] AND "percutaneous"[All Fields]) OR "percutaneous nephrolithotomy"[All Fields] OR ("percutaneous"[All Fields] AND "nephrolithotomy"[All Fields])) |
| ((((((body mass index) OR (BMI)) OR (obese)) OR (obesity)) OR (overweight)) AND (nephrolithiasis)) AND (treatment) | ("body mass index"[MeSH Terms] OR ("body"[All Fields] AND "mass"[All Fields] AND "index"[All Fields]) OR "body mass index"[All Fields] OR "BMI"[All Fields] OR ("obeses"[All Fields] OR "obesity"[MeSH Terms] OR "obesity"[All Fields] OR "obese"[All Fields] OR "obesities"[All Fields] OR "obesity s"[All Fields]) OR ("obeses"[All Fields] OR "obesity"[MeSH Terms] OR "obesity"[All Fields] OR "obese"[All Fields] OR "obesities"[All Fields] OR "obesity s"[All Fields]) OR ("overweight"[MeSH Terms] OR "overweight"[All Fields] OR "overweighted"[All Fields] OR "overweightness"[All Fields] OR "overweights"[All Fields])) AND ("nephrolithiasis"[MeSH Terms] OR "nephrolithiasis"[All Fields]) AND ("therapeutics"[MeSH Terms] OR "therapeutics"[All Fields] OR "treatments"[All Fields] OR "therapy"[MeSH Subheading] OR "therapy"[All Fields] OR "treatment"[All Fields] OR "treatment s"[All Fields]) |
| ((((((body mass index) OR (BMI)) OR (obese)) OR (obesity)) OR (overweight)) AND (urolithiasis)) AND (treatment) | ("body mass index"[MeSH Terms] OR ("body"[All Fields] AND "mass"[All Fields] AND "index"[All Fields]) OR "body mass index"[All Fields] OR "BMI"[All Fields] OR ("obeses"[All Fields] OR "obesity"[MeSH Terms] OR "obesity"[All Fields] OR "obese"[All Fields] OR "obesities"[All Fields] OR "obesity s"[All Fields]) OR ("obeses"[All Fields] OR "obesity"[MeSH Terms] OR "obesity"[All Fields] OR "obese"[All Fields] OR "obesities"[All Fields] OR "obesity s"[All Fields]) OR ("overweight"[MeSH Terms] OR "overweight"[All Fields] OR "overweighted"[All Fields] OR "overweightness"[All Fields] OR "overweights"[All Fields])) AND ("urolithiasis"[MeSH Terms] OR "urolithiasis"[All Fields]) AND ("therapeutics"[MeSH Terms] OR "therapeutics"[All Fields] OR "treatments"[All Fields] OR "therapy"[MeSH Subheading] OR "therapy"[All Fields] OR "treatment"[All Fields] OR "treatment s"[All Fields]) |
| ((((((body mass index) OR (BMI)) OR (obese)) OR (obesity)) OR (overweight)) AND (renal stone)) AND (treatment) | ("body mass index"[MeSH Terms] OR ("body"[All Fields] AND "mass"[All Fields] AND "index"[All Fields]) OR "body mass index"[All Fields] OR "BMI"[All Fields] OR ("obeses"[All Fields] OR "obesity"[MeSH Terms] OR "obesity"[All Fields] OR "obese"[All Fields] OR "obesities"[All Fields] OR "obesity s"[All Fields]) OR ("obeses"[All Fields] OR "obesity"[MeSH Terms] OR "obesity"[All Fields] OR "obese"[All Fields] OR "obesities"[All Fields] OR "obesity s"[All Fields]) OR ("overweight"[MeSH Terms] OR "overweight"[All Fields] OR "overweighted"[All Fields] OR "overweightness"[All Fields] OR "overweights"[All Fields])) AND ("kidney calculi"[MeSH Terms] OR ("kidney"[All Fields] AND "calculi"[All Fields]) OR "kidney calculi"[All Fields] OR ("renal"[All Fields] AND "stone"[All Fields]) OR "renal stone"[All Fields]) AND ("therapeutics"[MeSH Terms] OR "therapeutics"[All Fields] OR "treatments"[All Fields] OR "therapy"[MeSH Subheading] OR "therapy"[All Fields] OR "treatment"[All Fields] OR "treatment s"[All Fields]) |
